# Natural Polymorphisms in Mycobacterium tuberculosis Conferring Resistance to Delamanid in Drug-Naive Patients

**DOI:** 10.1128/AAC.00513-20

**Published:** 2020-10-20

**Authors:** Martina L. Reichmuth, Rico Hömke, Kathrin Zürcher, Peter Sander, Anchalee Avihingsanon, Jimena Collantes, Chloé Loiseau, Sonia Borrell, Miriam Reinhard, Robert J. Wilkinson, Marcel Yotebieng, Lukas Fenner, Erik C. Böttger, Sebastien Gagneux, Matthias Egger, Peter M. Keller

**Affiliations:** a Institute of Social and Preventive Medicine, University of Bern, Bern, Switzerland; b Institute of Medical Microbiology, University of Zurich, Zurich, Switzerland; c Swiss National Center for Mycobacteria, Zurich, Switzerland; d The HIV Netherlands Australia Thailand (HIV-NAT) Research Collaboration, Thai Red Cross AIDS Research Centre and Tuberculosis Research Unit, Faculty of Medicine, Chulalongkorn University, Bangkok, Thailand; e Instituto de Medicina Tropical Alexander von Humboldt, Universidad Peruana Cayetano Heredia, Lima, Peru; f Swiss Tropical and Public Health Institute, Basel, Switzerland; g University of Basel, Basel, Switzerland; h Wellcome Centre for Infectious Diseases Research in Africa, University of Cape Town, Cape Town, Republic of South Africa; i Department of Infectious Diseases, Imperial College, London, United Kingdom; j The Francis Crick Institute, London, United Kingdom; k National TB Lab, Kinshasa, Democratic Republic of the Congo; l Albert Einstein College of Medicine, New York, New York, USA; m Centre for Infectious Disease Epidemiology and Research, Faculty of Health Sciences, University of Cape Town, Cape Town, Republic of South Africa; n Institute for Infectious Diseases, University of Bern, Bern, Switzerland

**Keywords:** *Mycobacterium tuberculosis*, delamanid, resistance, mutations, drug resistance, natural polymorphism

## Abstract

Mutations in the genes of the F_420_ signaling pathway of Mycobacterium tuberculosis complex, including *dnn*, *fgd1*, *fbiA*, *fbiB*, *fbiC*, and *fbiD*, can lead to delamanid resistance. We searched for such mutations among 129 M. tuberculosis strains from Asia, South America, and Africa using whole-genome sequencing; 70 (54%) strains had at least one mutation in one of the genes. For 10 strains with mutations, we determined the MIC of delamanid. We found one strain from a delamanid-naive patient carrying the natural polymorphism Tyr29del (*ddn*) that was associated with a critical delamanid MIC.

## Abstract (precis)

Mutations in the genes of the F_420_ signaling pathway of Mycobacterium tuberculosis complex, including *dnn*, *fgd1*, *fbiA*, *fbiB*, *fbiC*, and *fbiD*, can lead to delamanid resistance. We searched for such mutations among 129 M. tuberculosis strains from Asia, South America, and Africa using whole-genome sequencing; 70 (54%) strains had at least one mutation in one of the genes.

## TEXT

In 2014, the new antituberculosis (anti-TB) drug delamanid (also known as OPC-67683, or Deltyba) was introduced (1). The World Health Organization (WHO) recommends the administration of delamanid if a standard effective drug regimen cannot be prescribed due to drug toxicity or resistance (2, 3). Thus, the European Medicines Agency (EMA) conditionally approved delamanid for the treatment of multidrug-resistant (MDR) TB (1, 3, 4). Of note, 6 years after its market launch, robust and widely accepted breakpoints that define susceptibility and resistance to delamanid still do not exist (5). The few available studies suggest a critical MIC between 0.125 mg/liter and 0.2 mg/liter, and an epidemiological cutoff value (ECOFF) of 0.04 mg/liter (6–9). This ECOFF is in line with the WHO technical report (10).

Delamanid is a drug of the bicyclic nitroimidazole class with potent anti-TB activity (1, 11). It is a prodrug that is activated by the deazaflavin (F_420_)-dependent nitroreductase (*ddn*) through hydride transfer, forming unstable intermediates, which in turn lead to the formation of reactive nitrogen species (nitric oxide, nitrous acid) (12, 13). Activated delamanid thus has a dual bactericidal mode of action: the primary decomposition product prevents mycolic acid synthesis, while the reactive nitrogen species cause respiratory poisoning (12–15). Loss-of-function mutations in *ddn* or one of the genes encoding the five coenzymes (*fgd1*, *fbiA*, *fbiB*, *fbiC*, and *fbiD*) have been proposed as a mechanism of resistance to delamanid (12, 13, 16, 17). *In vitro*, the frequencies of delamanid resistance-conferring mutations in the Mycobacterium tuberculosis laboratory strain H37Rv and in Mycobacterium bovis range from 2.51 × 10^−5^ to 6.44 × 10^−6^ (13). Previous studies have found several resistance-conferring mutations, including Leu107Pro (*ddn*), 51–101del (*ddn*), Trp88STOP (*ddn*), Gly81Asp (*ddn*), Gly81Ser (*ddn*), Gly53Asp (*ddn*), c.146_147insC (*fgd1*), Gln88Glu (*fgd1*), Lys250STOP (*fbiA*), Arg175His (*fbiA*), and Val318Ile (*fibC*) (6–8, 18–22).

This multicenter study has been described in detail elsewhere and is part of the work of the International epidemiology Databases to Evaluate AIDS (IeDEA) (23). We identified putative delamanid resistance-conferring mutations in M. tuberculosis strains from TB patients living with HIV (PLWH) and delamanid-naive, HIV-negative TB patients by whole-genome sequencing (WGS) and MIC determination. We collected information on the demographic and clinical characteristics of patients who were recruited between 2013 and 2016 in Peru, Thailand, Côte d’Ivoire, the Democratic Republic of the Congo (DRC), Kenya, and South Africa (24, 25). The Cantonal Ethics Committee in Bern, Switzerland, and local institutional review boards approved the study. Written informed consent was obtained at all locations, except in South Africa, where consent was not required for archived samples.

The sequencing pipeline has been described previously (25). In brief, M. tuberculosis DNA was extracted and sequenced using the Illumina HiSeq 2500 system (Illumina, San Diego, CA, USA). For the analysis, we used the well-established pipeline TBprofiler (https://github.com/jodyphelan/TBProfiler) (26, 27). It aligns short reads to the M. tuberculosis reference strain H37Rv (GenBank accession no. NC_000962.3) with bowtie2 (v2.3.5), BWA (v0.7.17), or minimap2 (v2.16) and then calls variants with SAMtools (v1.9) (28–31). To identify putative delamanid resistance-conferring mutations, we analyzed F_420_ genes (*ddn*, *fgd1*, *fbiA*, *fbiB*, *fbiC*, and *fbiD*) with variant frequencies of ≥75%. A subset of M. tuberculosis strains with at least one mutation in the F_420_ genes was recultured in liquid medium and subjected to delamanid MIC determination (see Fig. S1 in the supplemental material). We assumed that 0.04 mg/liter indicates a critical MIC (9).

We included 129 M. tuberculosis isolates, among them 51 isolates (39.5%) from Peru, 13 (10.1%) from Thailand, 49 (38%) from Côte d’Ivoire, 14 (10.9%) from the DRC, and 1 (0.8%) each from Kenya and South Africa. We identified 70 (54.3%) isolates with polymorphisms in at least one of the six F_420_ genes compared to the reference genome (Table S1). All patients infected with either of these strains were naive to delamanid. We selected strains fulfilling the following criteria: (i) mutations in a part of the gene encoding regions of catalytic or structural importance predicted by ARIBA and then the PhyResSE pipeline (32, 33), (ii) availability of a culture of the strain, and (iii) bacterial growth amenable to microdilution (25). MICs were determined for 10 isolates with mutations in the F_420_ genes. Four isolates showed MICs of >0.015 mg/liter: specifically, MICs of 0.5 (patient 1), 0.03 (patients 6 and 10), and >8 (patient 9) mg/liter (Table 1; Fig. S1). The isolate from patient 1 had a polymorphism in *fgd1* (Lys270Met) and was susceptible to the six drugs tested (isoniazid, rifampin, ethambutol, pyrazinamide, moxifloxacin, and amikacin). The patient was cured. The isolate from patient 9 had two alterations: a deletion in *ddn* (Tyr29del) and a nucleotide change in *fgd1* (T960C). The strain showed an elevated delamanid MIC and was phenotypically susceptible to six other drugs tested. The patient died. The MIC for the isolates of patients 10 and 6 was above 0.015 but below 0.04 mg/liter (Table 1). This suggests low-level resistance to delamanid (22), which could be due to the combination of various mutations: Ala416Val (*fbiC*), Trp678Gly (*fbiC*), Arg64Ser (*fgd1*), and T960C (*fgd1*).

**TABLE 1:** Observed polymorphisms in F_420_ genes and MIC values of delamanid^*a*^

Patient no. or reference	Lineage	Country	HIV status	Age (yr) at TB diagnosis	Gender	Mutation(s) in the F_420_ genes	Treatment outcome	MIC (mg/liter) in the microdilution
Reference	H37Rv (ATCC 27294)					Control (wt)		≤0.015
**1**	**L4.1.2.1**	**Côte d’Ivoire**	**Negative**	**29**	**Female**	***fgd1* Lys270Met**	**Cured**	**0.5**
2	L4.6.2.2	Côte d’Ivoire	Negative	51	Male	*ddn* C168T	Died	≤0.015
3	L2.2.1	Kenya	Positive	40	Male	*fgd1* T960C	Died	≤0.015
4	L2.2.1	Peru	Positive	28	Male	*fgd1* T960C	Unknown	≤0.015
5	L4.3.2	Peru	Negative	21	Male	*fbiC* C1161T	Cured	≤0.015
**6**	**L4.1.2.1**	**Peru**	**Positive**	**45**	**Male**	***fgd1* Lys270Met**	**Unknown**	**0.03**
7	L4.1.2.1	Peru	Positive	36	Male	*fbiC* G-11A, *fgd1* Lys270Met	Unknown	≤0.015
8	L4.1.2	South Africa	Negative	57	Female	*fbiA* Ile208Val	Cured	≤0.015
**9**	**L2.2.1**	**Thailand**	**Unknown**	**76**	**Male**	***fgd1* T960C, *ddn* 85-87del (Tyr29del)**	**Died**	**>8**
**10**	**L1.1.1**	**Thailand**	**Negative**	**42**	**Male**	***fbiC* Ala416Val Trp678Gly, *fgd1* Arg64Ser T960C**	**Unknown**	**0.03**

a All patients were treated with 2 months of daily isoniazid, rifampin, pyrazinamide, and ethambutol, followed by 4 months of daily rifampin and isoniazid. Data for isolates for which the MIC was >0.015 are shown in boldface. wt, wild type; L, lineage.

In summary, in the subset of 10 isolates with polymorphisms in the six targeted genes, six had no elevated MIC in the microdilution, while four isolates had elevated MICs (Table 1). In line with previous studies, we found that Lys270Met in *fgd1* is a natural polymorphism characteristic of M. tuberculosis lineage 4.1.2.1, which may (patients 1 and 6) or may not (patient 7) lead to an increased delamanid MIC (19, 34, 35). All 16 strains of lineage 4.1.2.1 showed this lineage-specific marker (Table S1). Furthermore, T960C (*fgd1*) is a synonymous substitution and was found in three other patient isolates which, as expected, did not have a critical MIC. The increase in the delamanid MIC for the isolate of patient 9 was due to the deletion in *ddn* (7). Our results thus suggest that Tyr29del is a natural polymorphism leading to an increased delamanid MIC. Our study was too small to estimate the prevalence of strains that are naturally resistant to delamanid. In 2020, Lee et al. screened 14,876 M. tuberculosis strains and found 2 strains with Tyr29del, for a prevalence of 0.013% (36). However, in their study, only the *ddn* gene was screened, and the prevalence of natural resistance could, therefore, be higher.

In conclusion, we confirm that mutations in F_420_ genes can confer an elevated delamanid MIC (13, 19). Whether our findings also apply to the related drug pretomanid should be investigated in future studies. The occurrence of clinical M. tuberculosis isolates from previously untreated patients for which delamanid MICs are naturally elevated calls for careful drug susceptibility testing (DST) prior to delamanid treatment (5, 36). However, access to DST is limited in high-burden countries. This dilemma highlights the conflict between making new drugs available in high-burden countries and avoiding the spread of drug-resistant strains.

### Data availability.

WGS data from patients’ M. tuberculosis strains shown in Table 1 have been submitted to the NCBI (BioProject accession no. PRJNA300846) (Table S1).

## Supplementary Material

Supplemental file 1

Supplemental file 2
