# Supplementary material for: Natural Polymorphisms in Mycobacterium tuberculosis Conferring Resistance to Delamanid in Drug-Naive Patients
Source: Antimicrob Agents Chemother. 2020 Oct 20;64(11):e00513-20. doi: 10.1128/AAC.00513-20 (PMC7577131; doi:10.1128/AAC.00513-20)
Supplement: Supplemental file 1 [file AAC.00513-20-s0001.pdf]

No.	DLM<br>8<br>mg/L	DLM<br>4<br>mg/L	DLM<br>2<br>mg/L	DLM<br>1<br>mg/L	DLM<br>0.5<br>mg/L	DLM<br>0.25<br>mg/L	DLM<br>0.125<br>mg/L	DLM<br>0.06<br>mg/L	DLM<br>0.03<br>mg/L	DLM<br>0.015<br>mg/L	GC	SC
**9**	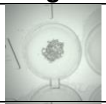	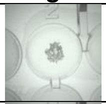	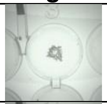	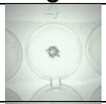	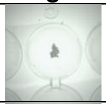	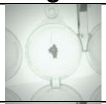	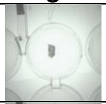	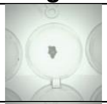	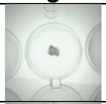	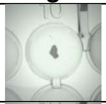	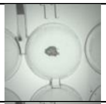	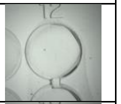
**5**	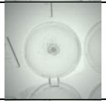	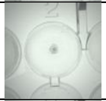	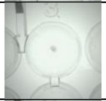	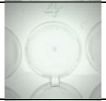	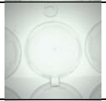	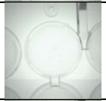	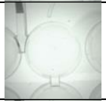	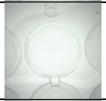	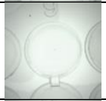	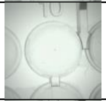	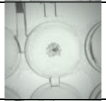	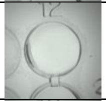
0	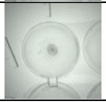	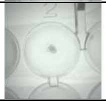	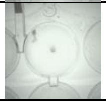	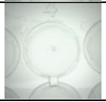	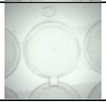	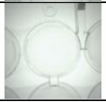	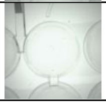	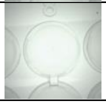	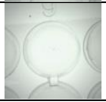	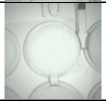	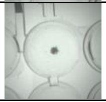	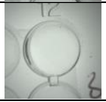

Supplementary Figure 1. *Mycobacterium tuberculosis* (*Mtb*) strains were pre-inoculated in Mycobacteria Growth Indicator Tubes (MGIT, BD, Franklin lakes, New Jersey, USA) and further processed at day 5 to 7 of positivity. The bacterial sediment was withdrawn from the MGIT tube, suspended in 1 ml MGIT broth, vigorously vortexed in the presence of glass beads (3 mm diameter) and further diluted 1:100. Then, a 96-well plate containing 50 µl of a ten-point-two-fold dilution series of delamanid, which was derived from dissolved tablets, was inoculated with 100 µl of the diluted bacterial suspension. The highest and lowest final delamanid concentration was 8 mg/L and 0.015 mg/L, respectively. Plates were sealed and incubated at 37 °C. Growth was documented by a microplate reader at days 7, 14 and 21. One well per strain without antibiotic served as positive growth control; one well without bacteria served as sterility control. Abbreviations: GC, growth control; SC, sterility control; DLM, delamanid; No., patient number; 0 = Reference strain H37Rv ATCC.
